# Physical activity in young children across developmental and health states: the *Active*CHILD study

**DOI:** 10.1016/j.eclinm.2023.102008

**Published:** 2023-05-25

**Authors:** Niina Kolehmainen, Christopher Thornton, Olivia Craw, Mark S. Pearce, Laura Kudlek, Kianoush Nazarpour, Laura Cutler, Esther Van Sluijs, Tim Rapley

**Affiliations:** a Population Health Sciences Institute, Newcastle University, Newcastle upon Tyne, UK; b School of Informatics, The University of Edinburgh, Edinburgh, UK; c MRC Epidemiology Unit, University of Cambridge School of Clinical Medicine, Cambridge Biomedical Campus, Cambridge, UK; d Department of Social Work, Education and Community Wellbeing, Northumbria University, Newcastle upon Tyne, UK

**Keywords:** Physical activity, Mobility, Child, Development, Long-term conditions

## Abstract

**Background:**

Evidence about physical activity of young children across developmental and health states is very limited. Using data from an inclusive UK cohort, ActiveCHILD, we investigated relationships between objectively measured physical activity, child development, social context, and health-related quality of life (HRQoL).

**Methods:**

Children (12–36 months), purposively sampled across health pathways, developmental abilities, and sociodemographic factors, were recruited through thirteen National Health Service organisations in England. Data were collected from 07/2017 to 08/2019 on: weekly physical activity (3–7 days) using waist-worn accelerometer (ActiGraph 3GTX); sociodemographics, parent actions, child HRQoL, and child development using questionnaires; and child health conditions using clinical records. A data-driven, unsupervised method, called hidden semi-Markov model (HSMM) segmented the accelerometery data and provided estimates of the total time spent active (any intensity) and very active (greater intensity) for each child. Relationships with the explanatory factors were investigated using multiple linear regression.

**Findings:**

Physical activity data were obtained for 282 children (56% females, mean age 21 months, 37.5% with a health condition) covering all index of multiple deprivation deciles. The patterns of physical activity consisted of two daily peaks, children spending 6.44 (SD = 1.39) hours active (any intensity), of which 2.78 (SD = 1.38) hours very active, 91% meeting WHO guidelines. The model for total time active (any intensity) explained 24% of variance, with mobility capacity the strongest predictor (β = 0.41). The model for time spent very active explained 59% of variance, with mobility capacity again the strongest predictor (β = 0.76). There was no evidence of physical activity explaining HRQoL.

**Interpretation:**

The findings provide new evidence that young children across developmental states regularly achieve mainstream recommended physical activity levels and challenges the belief that children with development problems need lower expectations for daily physical activity compared to peers. Advancing the rights of all children to participate in physical activity requires inclusive, equally ambitious, expectations for all.

**Funding:**

Niina Kolehmainen, HEE/NIHR Integrated Clinical Academic Senior Clinical Lecturer, NIHR ICA-SCL-2015-01-00, was funded by the 10.13039/100006662NIHR for this research project. Christopher Thornton, Olivia Craw, Laura Kudlek, and Laura Cutler were also funded from this award. Tim Rapley is a member of the NIHR Applied Research Collaboration North East and North Cumbria, with part of his time funded through the related award (NIHR200173). The views expressed in this publication are those of the author(s) and not necessarily those of the NIHR, NHS, or the UK Department of Health and Social Care. The work of Kianoush Nazarpour is supported by 10.13039/501100000266Engineering and Physical Sciences Research Council (EPSRC), under grant number EP/R004242/2.

**Research in context**

**Evidence before this study**

For the past decade, published systematic reviews have consistently concluded that: childhood physical activity, including under5s activity, is important for various health and developmental outcomes; objectively measured evidence about the under5s activity is too limited to allow effective policy and intervention development; and there is a need for more inclusion of disadvantaged groups, especially those with early health and/or developmental problems in the studies. Most of these reviews have also concluded that meta-analyses have not been possible, and that the quality of current evidence in under5s is from low to medium. Two of the latest evidence summaries are those that resulted in the UK Chief Medical Officers’ guidelines and that focused on people living with disability. The former concluded that the evidence-base used to develop guidelines for the under5s has largely been restricted to studies of apparently healthy, typically developing, individuals; the latter similarly noted the lack of inclusion of people with disability in mainstream studies as a key evidence gap.

**Added value of this study**

The present study, for the first time, objectively measures physical activity in very young children across abilities, with intersecting data on age, health, and deprivation. The findings provide new evidence that most young children across developmental states can, and regularly do, achieve mainstream recommended physical activity levels. This strongly challenges the belief that children with development problems need lower expectations for daily physical activity compared to their peers; and brings to question the drive to separate disability guidelines, for example by the WHO and the UK Chief Medical Officers, that set lower expectations for children with disabilities.

**Implications of all the available evidence**

Building on the thread initiated in the Lancet 2021, on the participation of people living with disabilities in physical activity, the findings from the present study suggest that to truly advance the rights of all children to participate in physical activity is likely to require inclusive, equally ambitious, expectations for all.

## Introduction

Everyday movement and physical activity promotes young children’s health, wellbeing, and biopsychosocial outcomes^1^, ^2^, ^3^ across children of health and developmental states.^4^, ^5^, ^6^, ^7^ Although the importance of early-life physical activity is widely recognised, for example, in the WHO (World Health Organisation) and national guidelines,^8^^,^^9^ children’s activity levels decline from as early as school entry,^10^^,^^11^ considerable uncertainty remains about preschool children’s physical activity,^12^^,^^13^ and effective early interventions remain scarce.^14^, ^15^, ^16^ There is now an urgent, widely agreed need for more robust and diverse evidence about early childhood physical activity^17^ to advance innovation, policy, and practice.

There are two particular gaps that require addressing. *First*, there is a need for objectively measured physical activity data which moves from the current focus on parental report to more precise quantitative estimates of activity using wearable technologies. Reliance on parent recall lacks granularity—young children’s physical activity happens cumulatively through short bursts that are difficult to subjectively estimate.^18^ Furthermore, as children spend time across nursery, school and home it is difficult for parents to accurately report on all of a child’s activity. *Second*, there is a need to include the currently underrepresented groups, especially children with developmental and health problems, in mainstream physical activity considerations. Much of current policy, practice, research, and laic discourse is built on an assumption that children with developmental or health problems are so different from their peers that they need to be treated and studied separately, and that population-level evidence and expectations do not apply to these children. They are often placed in specialist pathways and neglected from, or invisible in, universal health surveillance, policy, and guidelines. The specialist guidelines for them, where these exist, often reflect lower expectations compared to the mainstream guidance; potentially underpinned by scarcity of high-quality research.^7^ One example of this are the UK Chief Medical Officers’ physical activity guidelines. The mainstream under5s guideline,^9^ applicable to typically developing children only on the grounds that evidence for children with disabilities is lacking,^19^ recommends a minimum of 180 min of daily activity, while the disabled children and young people guideline^20^ recommends only 20 min of activity. Similarly, the WHO guidelines^8^ suggest differential treatment of children with health concerns compared to their peers. Considering the very real potential that differing policies and guidelines can further perpetuate inequalities in health,^21^ it is important to investigate the assumption that health behaviours in children with differing developmental and health states are, and need to be, radically different.

The ActiveCHILD study was set up to investigate movement and physical activity as an everyday health behaviour in young children across developmental abilities, and health states. The present paper reports on: (i) the levels and patterns of objectively measured physical activity in children 12–36 months; (ii) relationships between child physical activity behaviours, development, and sociobehavioural context; and (iii) relationship between child physical activity, and health related quality of life. The results provide new evidence to inform more inclusive policy and practice to promote physical activity across all children.

## Methods

### Design

The ActiveCHILD study recruited 349 children (age 12–36 months) from thirteen National Health Service (NHS) organisations in England, UK, and collected longitudinal data on children’s 7-day physical activity within everyday life. Full protocol and materials can be accessed online.^22^

The study drew on best practice guidance for health research involving children,^23^ including: supporting young children’s right to make a contribution; emphasising their right to shape the project and participate on their own terms; involving children and parents in designing the study; fairly representing the likely risks, burdens, and benefits; actively seeking to enable children and parents to make informed decisions throughout the research process; proactively building good relationships and trust to facilitate open communication; and giving children as much control over their participation as possible, including sensitivity to their preferences not to participate.

### Recruitment

We adopted a pragmatic approach to recruitment to include children with a range of abilities and backgrounds. All children attending their routine 12- or 24-month checks in the participating organisations, as part of the universal health pathway, were sent a study invitation from their health visiting service. These checks are intended for all children, however anecdotally children with developmental and health problems often do not receive them. As we had very few replies for these children in the first 6 months of the study we further invited children via a second, specialist-pathway. This focused on children seen at the participating pediatric community and outpatient services, and where there was either a raised or an established concern about the child’s physical capacity—operationalised in line with common clinical practice as a concern about body structure or functioning (motor, movement, growth, bones, joints, or muscles), where the underlying aetiology may or may not have been known, warranting a clinical referral. This second recruitment pathway was intended to ensure the diversity of children (on capacity and health status) within the sample, not to create two comparative cohorts for analysis. Children’s diagnoses were recorded but not used as a criterion, which reflects the realities of pediatric emerging diagnoses. The profile of the final, included sample of children is described in Results, below (Table 1), with a breakdown of the key characteristics for children recruited through each pathway also provided. In both the universal and specialist recruitment pathways, parents interested in the study contacted the study team by a postal return slip, phone or email, and if agreeable were consented by a researcher. The present analysis draws on baseline data from 282 children, born between 02/2015 and 07/2018, who provided eligible data, collected from 07/2017 to 08/2019.

**Table 1:** Participant characteristics at baseline across the total sample of participants returning usable accelerometer data, and split by recruitment pathway.

	Total	Universal pathway	Specialist pathway
Total number of children included	282	163	118
Child sociodemographic details			
Sex (% female) (n = 280)	56.4	55.5	58.1
Age in months (mean [SD]) (n = 281)	21 [8]	20 [8]	22 [7]
Language spoken at home (%) (n = 221)			
English	85.1	90.1	76.2
Non-verbal	6.8	2.8	13.8
Multilingual	4.5	5.0	3.8
Sign Language	0.9	0.0	2.5
Other^a^	2.7	2.1	3.6
Index of Multiple Deprivation decile (n = 277)			
1 [ = most deprived] (%)	13.4	8.8	19.5
2 (%)	10.5	8.2	13.6
3 (%)	7.9	6.3	10.2
4 (%)	6.9	3.1	11.9
5 (%)	7.9	7.5	8.5
6 (%)	9.0	10.1	7.6
7 (%)	13.0	17.0	7.6
8 (%)	5.8	5.0	6.8
9 (%)	13.0	16.4	8.5
10 [ = least deprived] (%)	12.6	17.6	5.9
Child health and development			
Born preterm (%) (n = 134)	23.9	11.4	41.8
Had a diagnosed medical condition (%) (n = 251)^b^	37.5	12.7	70.4
BMI-SDS at baseline^c^ (mean [SD]) (n = 105)	0.51 [1.19]	0.67 [1.07]	0.1 [1.39]
BMI at baseline (mean [SD]) (n = 115)	16.9 [1.6]	17.2 [1.5]	16.2 [1.8]
BMI-length or height (cm) (mean [SD]) (n = 115)	83.1 [7.6]	83.9 [6.6]	81.0 [9.6]
BMI – weight (kg) (mean [SD]) (n = 115)	11.6 [2.4]	12.0 [2.2]	10.9 [2.4]
Social-cognitive capacity^d^ (mean [SD]) (n = 252)	55.2 [4.8]	56.2 [4.4]	53.9 [5.0]
Mobility capacity^d^ (mean [SD]) (n = 252)	56.8 [6.6]	59.1 [5.0]	53.9 [7.2]
Walks without support (%) (n = 202)	80.0	95.4	62.5
Uses walking aid (%) (n = 202)	10.2	0.9	20.8
Meets PA guidelines at baseline (%) (n = 282)^e^	91	98	81
Environmental factors			
Main Carer Education (%) (n = 217)			
CSE below grade I (n = 4)	1.8	0.7	3.9
O’Level, GCSE grades A∗-C, CSE grade I (n = 24)	11.1	7.9	16.9
A’Level, Scottish Certificate of 6th year studies, SCE Higher, AS level (n = 22)	10.1	9.3	11.7
Diploma in Higher Education (n = 15)	6.9	6.4	7.8
First Degree, Higher Degree (n = 152)	70.0	75.7	59.7
Main Carer Weekly Work Hours (median, [IQR])	18 [30]	20 [30]	11 [24]
Time to safe outdoors place in minutes (median [IQR]) (n = 217)	10 [10]	10 [5]	10 [14]
Transport method to the safe outdoors place (n = 147)			
Car (%)	23	20.6	25
Public Transport (%)	2	1.4	4
Bicycle (%)	3	4.2	1
Foot (%)	85	87.9	79

SD = standard deviation; IQR = interquartile range; kg = kilograms; CSE = Certificate of Secondary Education; GCSE = General Certificate of Secondary Education; SCE = Scottish Certificate of Education.

a Arabic, Bengali, Bulgarian, Danish, Polish, Punjabi, Spanish.

b The full break down of diagnostic categories is available online [https://doi.org/10.25405/data.ncl.21407457].

c Standardised (zscore) Body Mass Index (BMI) calculated using the anthro R package which implements the guidance set *out in recommendations for data collection, analysis and reporting on anthropometric indicators in children under 5 years old*.

d Pediatric evaluation of disability inventory computer adaptive testing (PEDI-CAT) subscale score.

e Calculated from accelerometer data using cut points (40 counts per 5 s) from Oftedal^24^ and World Health Organisation (WHO) guidelines of 180 min of physical activity per day.

### Outcomes

**Fig. 1:**
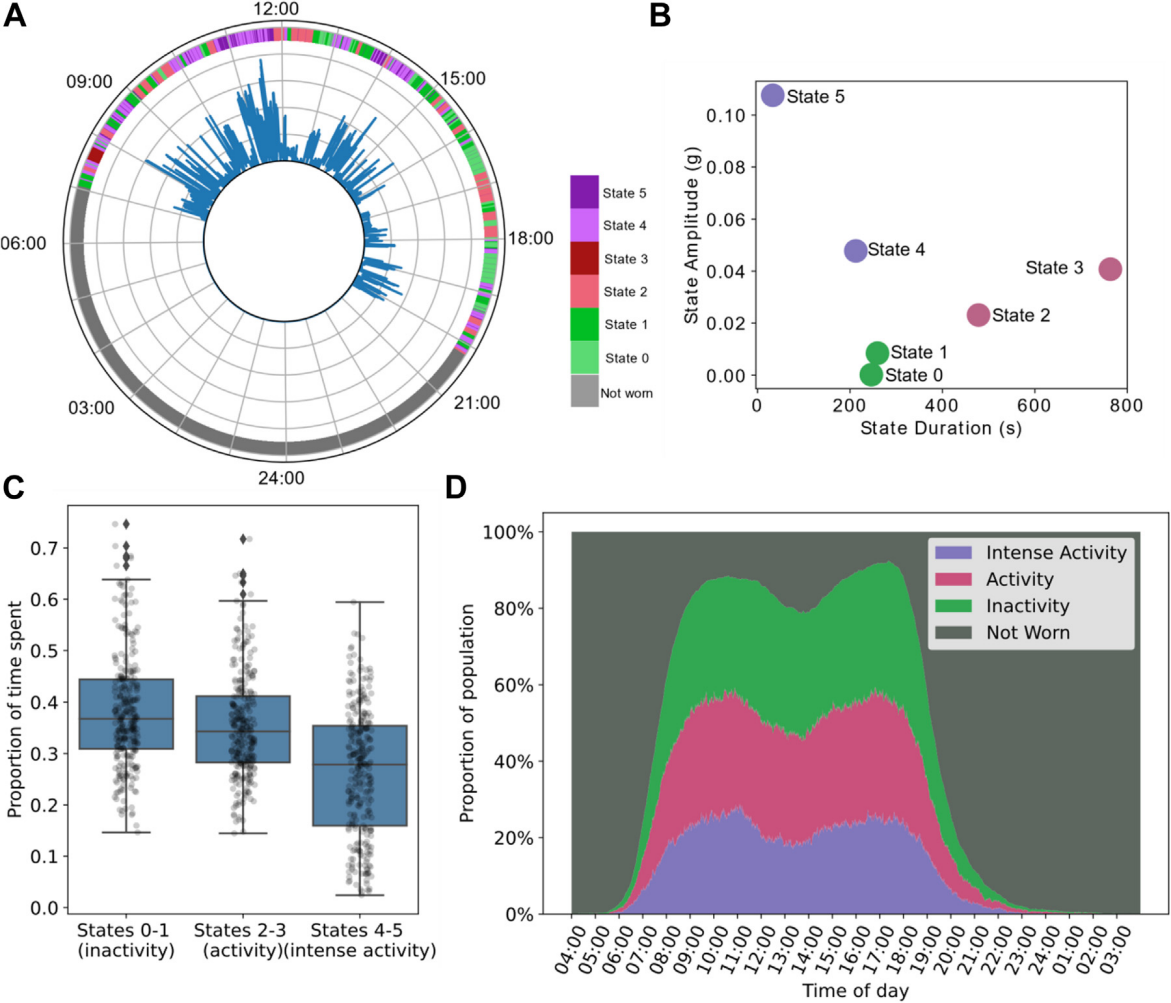
**Participation in activity intensity categories across the sample.** (A) An illustration of the acceleration recorded from one child in a single day, including the acceleration magnitude (the inner blue line) and the HSMM (Hidden Semi-Markov Model) state that has been assigned to that portion of the trace (the outer coloured bar). (B) The parameters of the HSMM states, including the mean of Gaussian distribution modelling the magnitude of the acceleration (the y axis) and the λ of the Poisson distribution modelling the duration of the state (the x axis). (C) The proportion of time spent in the three activity intensity categories across the cohort with each point representing the proportion of time spent by each child. (D) Distribution of physical activity across the day and activity states across the sample.

The primary outcome of physical activity was measured using an accelerometer, the ActiGraph GT3X+, set to record all movement that lasted at least 1 s. The ActiGraph GT3X+ has been found acceptable to use by under5s and their parents across developmental capacities. Parents were asked to place the monitor on their child’s hip or lower back and wear it for seven consecutive days during waking hours, except water-based activities. Steps were taken to maximise good quality data returns (see protocol^22^). Non-wear time was defined as any continuous period of minimal movement longer than 1 h. Up to seven consecutive days that provided the maximum amount of wear time were included in the analysis. A recording day was included if it contained at least 5 h of wear time, and the overall recording included if it contained at least three such days. From the included recordings, the time spent inactive, active, and highly active were extracted using the hidden semi-Markov model (HSMM) approach.^25^^,^^26^ This classified each part of the acceleration trace into states 0–5, indicating the activity intensity at that time. Fig. 1A illustrates the acceleration recorded on a typical day, along with the HSMM state assigned to each segment of the day, while Fig. 1B displays the mean acceleration amplitude, and duration of each HSMM state. The clustering of the states was used for the further analysis, by grouping them. States 0 and 1 with the lowest amplitude were considered to reflect inactivity, states 2 and above are considered to reflect activity, and states 4 and 5 considered to reflect intense activity. For subsequent analysis we considered the time spent in states 2 to 5 as the total time spent active, and time in states 4–5 as time in intense activity. We calculated these as a proportion of the total time that the device was worn to account for the variance in the duration of wear time. A cut points approach was also used to calculate the time spend by children physically active (40 counts per minute^27^), allowing us to calculate the number of children meeting the WHO advised guidelines.

The secondary outcome of parent-reported child health-related quality of life (HRQoL) was measured using the Pediatric Quality of Life Inventory (PedsQL 4.0), a widely used, generic 23-item instrument for children age 2–18 years across abilities.^28^ Total scores were calculated, ranging from 0 to 4, with 0 indicating the lowest and 4 the highest HRQoL.

### Measures: explanatory factors and sample characteristics

Factors related to the child, their family, and physical environment were assessed as potential determinants (Supplementary Table S1). For child-factors, child’s sociodemographic data (sex, date of birth, postcode, language spoken at home) were collected using a parent questionnaire. Child’s height/length, weight, and formal medical diagnoses were extracted from NHS notes by healthcare provider and provided to the research team. Height/length and weight were used to calculate BMI Standard Deviation Scores (SDS) using the WHO guideline. Medical diagnoses and language spoken at home were categorised iteratively. Date of birth and the first date of accelerometer data were used to calculate age at data collection. Postcode was used to assign a rank from the Index of Multiple Deprivation (IMD) 2015 at Lower-layer Super Output Area (LSOA) level. LSOA’s are small areas of similar population sizes (an average of approximately 1500 residents or 650 households in each area) and produced in England by the Office for National Statistics. The LSOAs are ranked from IMD 1 (most deprived) to IMD 32,844 (the least deprived). IMD ranks were calculated into 10 IMD deciles (1 = most deprived, 10 = least deprived), based on the deciles for England, and these deciles reported. Child motor and social-cognitive capacity were measured using two domains of the Pediatric Evaluation of Disability Inventory Computer Adaptive Test (PEDI-CAT): mobility (e.g. ‘Stands for a few minutes’), and social/cognitive (e.g. ‘Uses single words, gestures, or signs to show what he/she wants’). PEDI-CAT^27^ is one of the few measures that has been explicitly designed and tested for use across diverse populations of children, both in terms of children’s ages and capacities. It is has been widely used in pediatric research, and has cumulative evidence both in terms of validity and reliability from a range of studies conducted by a number of research groups (an example list of key papers can be found at https://www.pedicat.com/publications/). Researchers completed the PEDI-CAT with parents at the baseline using an iPad, either in person at the initial recruitment visit or over the phone, depending on parent preference. PEDI-CAT scaled scores (i.e. the raw domain scores) were used for the analysis. The scaled scores are not adjusted for age, but we included age as a co-variate. This is the recommended approach by the developer, and was considered methodologically acceptable given the narrow age range of the children. The recruitment pathway (universal, specialist) was noted for each child, as a proxy of whether the child was, at the system-level, treated as mainstream or as within a clinical population.

Sociobehavioural context was operationalised as parent actions to facilitate, regulate, limit, or encourage the child’s movement and physical activity in the last 7 days. These were measured using a study-specific, online parent questionnaire comprising 19 items developed from existing qualitative literature, identified using a systematic search and screen.^29^ The questionnaire asked parents about the frequency at which they had taken specific actions in the past week. Physical environment was measured as parent-reported distance to safe outdoor play, and weather data extracted from the UK MetOffice database using regional weather station proximal to the child’s postcode.

Data were described using mean and standard deviation (continuous, normally distributed data), median and interquartile range (skewed, continuous, and ordinal data) or proportions (binary data). IBM SPSS Statistics version 21 (IBM Corp, Armonk, New York), STATA 17 (StataCorp), Python, R, and Excel were used for data management and analysis.

### Statistical analysis

For physical activity, we first used univariate Spearman’s rank correlation to explore relationships between all potential explanatory factors and the proportion of time spent active (model 1) or the proportion of time spent doing intense activity (model 2). For each model, we then used multiple linear regression to explore the relationships between physical activity and parent actions with the PEDI-CAT mobility score as a covariate to allow for a possibility that parent actions depended on the child’s capacity. Any variables significant at a p-value of ≤0.2 in the initial exploratory analyses were taken forward for the subsequent multivariable analysis. This less stringent criterion value was used to minimize the rate of false-negative results. The multivariable analysis consisted of a multiple linear regression model with all variables identified as predictors and physical activity participation (either total time active or intensely active) as the outcome. Before regression, all variables were standardised so that the variance of independent and dependent variables was equal to 1, resulting in standardised coefficients. Participants who did not have data for all variables were excluded from the multiple regression.

For HRQoL, we similarly first used univariable analysis (see above), followed by a multiple linear regression model with PedsQL (calculated as the mean of all contributing scores) as the dependent variable and the key child characteristics (age, IMD, mobility, social/cognitive capacity, and recruitment pathway) as well as the total time spent active as the pre-specified independent variables. Coefficients, confidence intervals, and p-values are reported for the regression results.

### Ethics statement

The study had NHS Research Ethics Committee and Health Regulation Authority approvals (Reference IRAS 218313, 17/NE/0051). A parental informed consent was obtained, and the best practice guidance for research involving children applied, as outlined above.

### Role of the funding source

The funder (National Institute for Health and Care Research, Engineering and Physical Sciences Research Council) had no involvement in study design; in the collection, analysis, and interpretation of data; in preparing the dataset; in the writing of the report; and in the decision to submit the paper for publication. We confirm that several authors (NK, CT, OC, LK, LC) directly accessed and verified the underlying data reported in the manuscript; access to anonymised data has also been made accessible for reviewing and research use at https://doi.org/10.25405/data.ncl.21120457. All authors (NK, CT, OC, MP, LK, KN, LC, EVS, TR) had final responsibility for the decision to submit for publication.

## Results

Of the 7100 recruitment packs provided to the recruiting clinical services, estimated 3000–6000 were sent out. These resulted in 648 responses, of which 181 declined to participate, 104 were interested but ultimately could not be contacted, and 358 were recruited, and provided with accelerometers. Of them, 305 wore and returned an accelerometer, of which 282 (92%) had recordings eligible for analysis. In 14 cases the parents reported the child declined to wear the accelerometer, in the remaining cases the reasons related to family circumstances or were unreported.

The 282 included children: had a mean age 21 months (sd = 8); were evenly distributed across sexes; covered all deciles of IMD; included children from across home-languages as well as non-verbal children; and included children with a range of health conditions (Table 1). Children recruited through the specialist pathway (118/282, 42%) were more likely to: live in more deprived areas; have a main carer without a university degree; have a language other than English spoken at home; report a longer distance to a safe outdoor place; have a medical diagnosis; have mobility limitations; and be non-verbal.

### Physical activity

On average, the children (n = 282) wore accelerometers for an estimated 10.43 (SD = 1.3) hours per day, of which they spent 6.44 (SD = 1.39) hours in any form of physical activity (states 2–5), including 2.78 (SD = 1.38) hours of intense activity (states 4 and 5). This corresponds to children spending 62% (SD = 11%) of their wear time active, including 29% (SD = 5%) of wear time in intense activity (Fig. 1C). Children recruited through the specialist pathway were less likely to meet the WHO physical activity guidelines (81% met guidelines) compared to children recruited through the universal pathway (98%).

In terms of daily patterns, most children first wore the accelerometers between 07:09 and 10:09, and last between 18:18 and 20:33. Between these times, inactivity, activity, and intense activity all increased rapidly though there was a dip in the middle of the day where non-wear time increased—likely attributable to the device being removed for nap times. Few children wore the accelerometers between 22:00 and 06:00, and the start and end times for a day showed limited variation across age groups (Supplementary Table S2). Fig. 1D shows the distribution of wear-time and physical activity intensities throughout the day.

### Parent actions

**Fig. 2:**
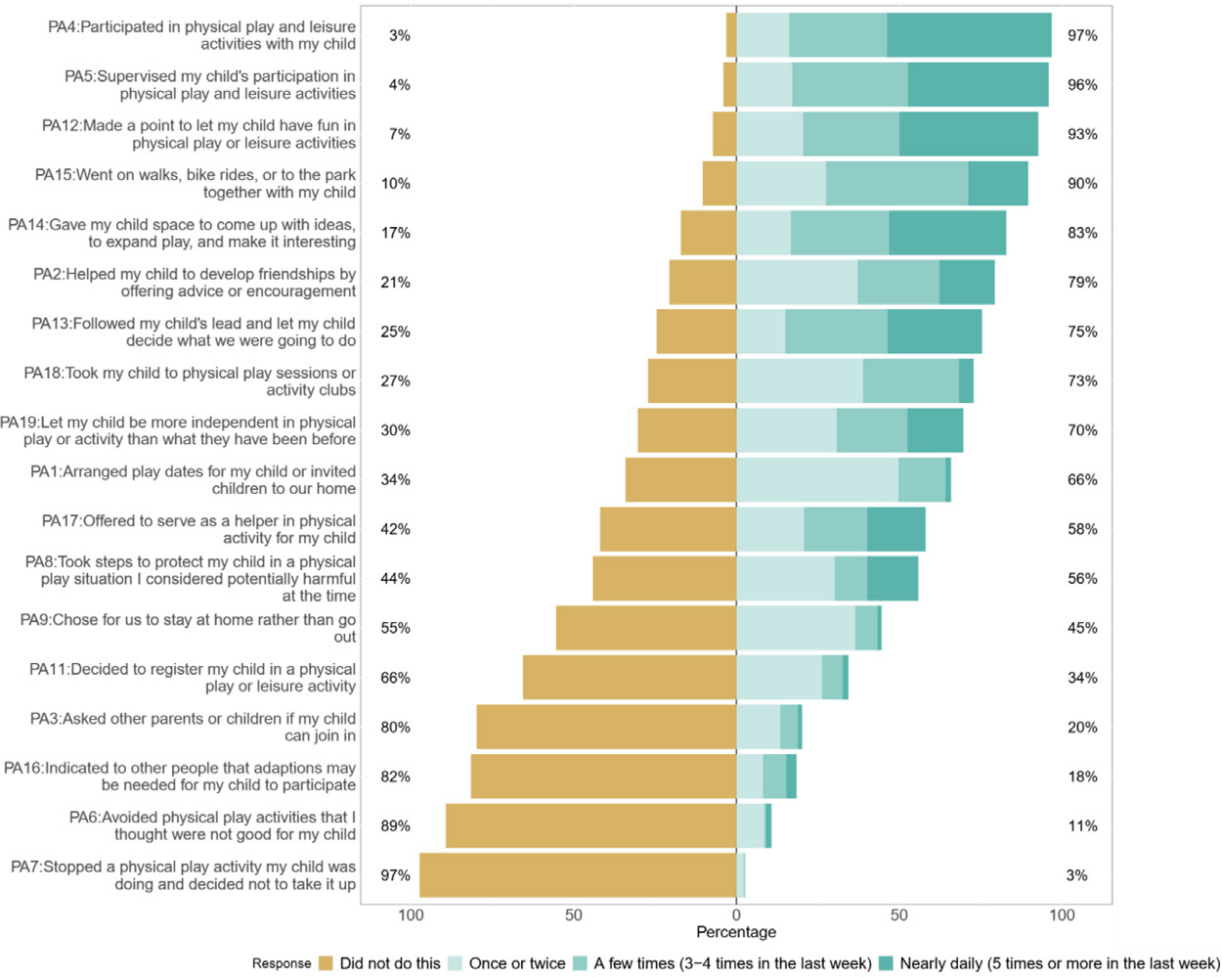
**Frequency of actions parents reported taking to facilitate, regulate, or limit their child’s physical activity**.

The most commonly reported actions to facilitate a child’s physical activity were (Fig. 2): participate in physical activities with the child; supervise the child’s participation in physical activity; making a point to let the child have fun in physical activities; and going for walks, bike rides or to the park with the child. One action potentially limiting physical activity was commonly reported: choosing for the family to stay at home rather than go out.

### Factors explaining physical activity

From the univariate analysis, age, recruitment pathway, sex, social-cognitive capacity, mobility, and main carer work hours were correlated with total time active (states 2–5). Age, IMD, recruitment pathway, social-cognitive capacity, mobility, travel time to an outdoor area, main carer work hours, and daily temperature were correlated with intense activity (states 4–5). From the exploratory analysis of parent actions (Supplementary Table S3), child mobility, physical activity, three actions (PA1 arranging play dates, PA2 helping child to develop friendships, and PA8 protecting child from risks and harm) were positively correlated with total time active and intense physical activity, and one (PA9 choosing to stay at home rather than go out) negatively correlated with both. Further three actions were positively correlated with intense physical activity: PA6 avoiding activities not good for the child; PA12 making a point to let the child have fun; and PA15 going on walks, bike rides, or to the park with the child. A further exploration of the variables taken forward for multivariable regressions are presented in Supplementary Tables S3 and S4 and Supplementary Figs. S1–S3.

Overall, the multivariable regression model of total time spent active (states 2–5), with eleven independent variables, explained 24% of variance (Table 2). The strongest predictor was child’s mobility (β = 0.41, 95% CI from 0.15 to 0.66), its structural coefficient indicating that it accounts for 72% of the final model. Male sex was a negative predictor (β = −0.14, CI from −0.27 to 0.01), as was the parent action of choosing to stay at home (β = −0.17, CI from −0.31 to −0.03). Further exploration showed that choosing to stay at home independently explained 11% (r_s_^2^ = 0.11) of the model output while sex showed a weaker relationship (r_s_^2^ = 0.05), suggesting that, although statistically significant in the present analysis, sex may have limited overall contribution to time active.

**Table 2:** The multivariable linear regression models of the selected independent variables and the proportion of time spent active (states 2 to 5) [Model 1].

Independent variable	Model 1: total time active (states 2–5) multivariable (n = 196)			
	Multiple linear regression (R^2^ = 0.24, Adj = 0.20)			
	p	β	95% CI	r_s_^2^
Recruitment pathway	0.55	−0.05	[−0.20, 0.11]	0.19
Age	0.87	−0.02	[−0.20, 0.17]	0.12
Mobility (PEDI-CAT)	**0.002**	**0.41**	[**0.15, 0.66]**	**0.72**
Social-cognitive (PEDI-CAT)	0.97	−0.01	[−0.28, 0.27]	0.41
Sex	**0.04**	**−0.14**	[**−0.27, 0.01]**	**0.05**
IMD (Decile)	0.25	0.08	[−0.06, 0.22]	0.09
Main carer work hours	0.55	−0.04	[−0.18, 0.10]	0.07
Parent Action 1 I arranged play dates for my child…	0.28	0.08	[−0.06, 0.21]	0.06
Parent Action 2 I helped my child to develop friendships…	0.65	0.03	[−0.10, 0.17]	0.09
Parent Action 8 I took steps to protect my child…	0.09	0.11	[−0.02, 0.25]	0.00
Parent Action 9 I chose for us to stay at home rather than go out.	**0.02**	**−0.17**	[**−0.31, −0.03]**	**0.11**

IMD = Index of Multiple Deprivation; PEDI-CAT = Pediatric Evaluation of Disability Inventory Computer Adaptive Testing.

Bold indicates significant at a p-value of <0.05.

The multivariable regression model of total time in intense activity (states 4–5), with fifteen independent variables, explained 59% of variance (Table 3). As with total time spent active, mobility was again the strongest predictor of time spent participating in intense activity (β = 0.76, 95% CI from 0.57 to 0.96), its structural coefficient indicating that it can account for 89% of the final model. Child’s age was a positive predictor (β = 0.16, CI from 0.02 to 0.30), as was the parent action of going on walks, bike rides, or to the park with the child (β = 0.15, CI from 0.04 to 0.27).

**Table 3:** The multivariable linear regression models of the selected independent variables and the proportion of time spent in intense activity (states 4 to 5) [Model 2].

Independent variable	Model 2: the proportion of time spent in intense activity (states 4 to 5) multivariable (n = 190)			
	Multiple linear regression (R^2^ = 0.59, Adj = 0.56)			
	p	β	95% CI	r_s_^2^
Recruitment pathway	0.80	0.01	[−0.10, 0.13]	0.10
Age	**0.03**	**0.16**	[**0.02, 0.30]**	**0.35**
Mobility (PEDI-CAT)	**<0.001**	**0.76**	[**0.57, 0.96]**	**0.89**
Social-cognitive (PEDI-CAT)	0.20	−0.14	[−0.35, 0.08]	0.58
IMD (Decile)	0.33	0.05	[−0.05, 0.16]	0.06
Time to outdoors	0.72	0.02	[−0.08, 0.12]	0.03
Main carer work hours	0.75	−0.02	[−0.13, 0.09]	0.03
Maximum daily temperature	0.60	−0.03	[−0.13, 0.08]	0.03
Parent Action 1 I arranged play dates for my child or invited children to our home	0.36	0.05	[−0.05, 0.15]	0.03
Parent Action 2 I helped my child to develop friendships…	0.11	0.08	[−0.02, 0.19]	0.07
Parent Action 6 I avoided physical play and leisure activities that I thought were not good for my child…	0.15	0.08	[−0.023, 0.18]	0.03
Parent Action 8 I took steps to protect my child in a physical play or leisure situation I considered risky or potentially harmful at the time…	0.66	0.02	[−0.08, 0.13]	0.02
Parent Action 9 I chose for us to stay at home rather than go out	0.66	−0.02	[−0.13, 0.09]	0.02
Parent Action 12 I made a point to let my child have fun in physical play or leisure activities	0.69	0.02	[−0.09, 0.13]	0.02
Parent Action 15 I went on walks, bike rides, or to the park together with my child	**0.01**	**0.15**	[**0.04, 0.27]**	**0.06**

IMD = Index of Multiple Deprivation; PEDI-CAT = Pediatric Evaluation of Disability Inventory Computer Adaptive Testing.

Bold indicates significant at a p-value of <0.05.

In both models social-cognitive capacity showed a strong structural coefficient but did not contribute significantly to the regression equation because the social-cognitive capacity and mobility were correlated (rho = 0.8), and as a result share explained variance. Overall, the models did not show strong multicollinearity (condition number = 4.51 for total time spent active, and condition number = 4.79 for time in intense activity).

### Health related quality of life

One hundred children in the cohort provided sufficient responses to compute a HRQoL score: a median of 0.76 (IQR = 0.46–1.3). While, in the univariable analysis, time spent active showed a negative association with HRQoL, this was not sustained in multivariable analysis (Supplementary Table S5). We consider the subset of 100 children with HRQoL data to be broadly representative of the overall sample: the children with HRQoL scores had a median IMD of 6 (the full sample n = 282 also median 6); were 55% female (56% in the full sample); 68% had a parent with a degree (60% in full sample); and 65% (58% in full sample) were from the universal pathway.

## Discussion

The ActiveCHILD study seeks to advance evidence about everyday movement and physical activity in children across developmental and health states. The baseline results show that young children spend, on average, 6 h per day in some form of movement activity, including just under 3 h of intense physical activity, with 9 in 10 children meeting current mainstream physical activity guidelines. Mobility (fundamental movement) capacity emerged as a key predictor of both the total and intense physical activity, and going outdoors also emerged as a predictor. There was little evidence that children’s physical activity relates to their health-related quality of life.

In terms of limitations, while all effort was made to avoid bias in sampling (e.g. inviting every eligible child within a region), the above-national-average educational level of the included parents is a potential risk to representativeness. We explored this in the analysis: educational level was not found to relate to physical activity. Also, like all studies, our findings are situated within context—care needs to be taken when seeking to extrapolate to other populations. It is also widely noted that the cut-points approach encounters issues when used with pre-school age children,^18^ motivating our use of the data-driven HSMM approach for most of our analyses. However, as physical activity recommendations are specified using cut-point categories, we opted to use the traditional approach here to enable compatibility. It is important to recognise missing data—we only had BMI data for 105 children and so this was not included in the final model, while missing data in other domains resulted in the exclusion of participants from the final multi-variable analysis. However, we have no reason to believe the missing data is related to any unobserved variables. We also did not collect data on the children’s experiences of care, and so were not able to consider the role of these in the analyses. Key strengths of the study included: objective measurement of physical activity with substantial wear times (average of 10 h per day); inclusion of a diverse sample of children across developmental and health states; and coverage of diverse geographical areas, and socioeconomic levels.

By using intersecting data on physical activity, age, health, developmental capacity, and deprivation, the results expand existing literature^10^^,^^11^^,^^30^ to show that inequalities in physical activity exist already in very early childhood, and that these inequalities are more likely to relate to child developmental capacity than sex or socioeconomic context. The results strongly indicate that young children with developmental challenges are less likely to experience movement activities than their peers. However, 81% of children recruited via specialist pathways met WHO physical activity guidelines, indicating that provided with an opportunity to be active at least 4 in 5 children with disabilities can meet, and exceed, current mainstream physical activity expectations.

This has important implications for policy and under5s physical activity guidelines. The findings suggest that the stated expectations for physical activity can be the same for all children—to date, there is no evidence to support an assumption that expectations for children with developmental problems or disabilities need be lower than for their peers. Given the importance of physical activity on present and long-term health and development, and the potential of guidelines to drive policy and practice norms, and resource allocation, setting unnecessary low expectations for children with developmental problems has the very real potential of further compounding inequalities. We are depriving the children from opportunities to promote their health and development, and prevent secondary problems, through physical activity. Furthermore, the findings from the present study add to the evidence that most young children meet the current physical activity recommendations,^30^ suggesting that expectations for all children could be more ambitious. Ensuring the expectations for all children are sufficiently high is an opportunity to promote major health benefits to the individuals, as well as economic benefits to the NHS and society.

Given the strong relationship between fundamental movement capacity and physical activity, further work is needed to better understand the drivers, mediators, and interactions explaining that relationship, as well as to explore different types of physical activities that children engage. This evidence would further help to identify specific adaptations that may be required to guidelines in order to make them inclusive across children with heterogenous demographics. In challenging the assumption that children with mobility limitations are unable to be active, the ActiveCHILD study invites us to explore further explanations for why they are less likely to be active than peers. Such work should focus on understanding the wider context and dynamic interactions within that, beyond parental actions.^31^ This is likely to require further work on quantification of young children’s everyday physical activity in ways that is accurate and practical as well as useable at scale, and that ultimately allows meaningful linkage of parental estimates of physical activity to guideline recommendations.

In conclusion, young children across developmental states can, and regularly do, achieve recommended physical activity levels. Assuming lower physical activity expectations for children with developmental problems than their peers is not justifiable in policy, practice or guidelines. To truly advance the rights of all children to participate in physical activity,^7^ physical activity guidelines and action plans need to be based on inclusive, equally ambitious, expectations for all.

## Contributors

Kolehmainen, Rapley, Van Sluijs, and Pearce conceived and designed the study. All authors made a substantial contribution to the acquisition and/or analysis and interpretation of the data. Thornton and Nazarpour developed the machine learning algorithm used to analyse the data. All authors drafted the work or substantively revised it, have approved the submitted version, agreed to be personally accountable for their contributions, and agreed to ensure that questions related to the accuracy or integrity of any part of the work, even ones in which the author was not personally involved, are appropriately investigated, resolved, and the resolution documented in the literature.

## Data sharing statement

The datasets used during the current study are available from the corresponding author on reasonable request. Anonymised data, a data dictionary, study protocol, and recruitment packs are also accessible for reviewing and research use at the Newcastle University data repository data.ncl and can be located using the following link: https://doi.org/10.25405/data.ncl.21120457.

## Declaration of interests

The authors declare that they have no competing interests.
