# Supplementary material for: Physical activity in young children across developmental and health states: the ActiveCHILD study
Source: eClinicalMedicine. 2023 May 25;60:102008. doi: 10.1016/j.eclinm.2023.102008 (PMC10220310; doi:10.1016/j.eclinm.2023.102008)
Supplement: Supplementary Tables S1–S5 and Fig. S1–S3 [file mmc1.docx]

**SUPPLEMENTAL FILE 1**

Table S1. Sample characteristics and potential explanatory factors, the measures used, and response options

| Factor | Measures | Response options |
| --- | --- | --- |
| Child’s functional status (mobility, social-cognitive, and self-care) | Pediatric Evaluation of Disability Inventory Computer Administered Testing (PEDI-CAT). | For the mobility and social/cognitive domains the 4-point response scale to the question stem “Can your child [do activity x]?” is: Unable; A little hard; Hard; Easy; I don’t know. |
| Child Mody Mass Index (BMI) | Weight, height/length converted to BMI-SDS using the World Health Organisation guidelines. | Free-text numbers. |
| Child health conditions and history | Confirmed medical diagnoses at baseline, walking ability and aids, preterm birth. | Extracted from National Health Service (NHS) case notes, yes/no responses with further free-text response to list any diagnoses. |
|  | Confirmed medical diagnoses after baseline, preterm birth. | Parent-report, yes/no responses with further free-text response to list any diagnoses. |
| Child sociodemographic factors | Child date of birth (DOB), sex, Postcode, language spoken at home. | Study-specific questionnaire, completed by parent online, using Qualtrics.  Response options:  *DOB: dd, mm, yyyy*  *Sex:* boy; girl; other.  *Main carer:* Mother; Father; Grandparent; Other.  *Main carer’s highest educational level:* free text, categorised for analysis as: CSE below grade 1; O’level, GCSE grades A*-C, CSE grade 1; A’ Level, Scottish Certificate of 6^th^ Year Studies, SCE Higher, AS Level; Diploma in Higher Education; or First Degree, Higher Degree.  *All other response options:* free text. |
| Family factors | Main carer, their educational status, and weekly hours worked. |  |
|  | A study-specific parent questionnaire (Adult Actions Questionnaire) consisting of 19 parent actions that have previously been reported, in qualitative literature, to influence children’s participation in physical activity. | Parent-report on how frequently they had taken an action in the last week, response options ranging from not done it at all to once or twice, a few times (3-4 times in the last week), to nearly daily (5 times in the last week or more. |
| Physical environment | Distance to a safe and usable outdoor play area as reported by parents through a parent questionnaire. | Parent self-report, open text response to the following: ‘How long does it take you go to the closest safe and usable outdoor play area for your child’ (responses provided in minutes/hours, and the mode of the transport selected from drop-down) |
|  | Weather as temperature (in degrees of Celsius) in the child’s area during the data collection, accessed through the UK Met Office. |  |

CSE = Certificate of Secondary Education; GCSE = General Certificate of Secondary Education; SCE = Scottish Certificate of Education.

**Table S2: The times that children put the device on in the morning and take it off at night.**

| **Age range (Number of children)** | **Mean time device first worn (standardised)** | **Mean time device last taken off (standardised)** |
| --- | --- | --- |
| 5 – 10 Months (n=13) | 07:47 (38 minutes) | 19:28 (70 minutes) |
| 10 – 15 Months (n=77) | 07:48 (63 minutes) | 19:18 (55 minutes) |
| 15 – 20 Months (n=49) | 07:53 (67 minutes) | 19:46 (98 minutes) |
| 20 – 25 Months (n=46) | 07:54 (72 minutes) | 19:43 (89 minutes) |
| 25 – 30 Months (n=61) | 07:55 (55 minutes) | 19:36 (62 minutes) |
| 30 – 35 Months (n=30) | 08:14 (86 minutes) | 19:28 (65 minutes) |
| 35 – 40 Months (n=2) | 09:49 (7 minutes) | 20:45 (7 minutes) |

Table S2 shows the mean times of the day that children, grouped by age, first wore the device in the morning and the latest before they took it off at night. Some children also took the accelerometer off during the day and put it back on again, in this case it would be the final time that contributes to this table. We see similar first worn and last taken off times across the age range, other than the oldest category where only two children contribute, and a large variance (around an hour) within each age group.

**Table S3. Variables included in multivariable regression analysis from the exploration of relationships between parent actions, child mobility, and child physical activity.**

|  |  |  | **Regression Equation**  **Y = β*Mobility (PEDI-CAT) + β*X + I** | |
| --- | --- | --- | --- | --- |
|  | **Parent Action (X)** | **Parent median response** | **β (Y -> Time active)** | **β (Y -> Time intensely active)** |
|  | 1 I arranged play dates for my child or invited children to our home. (n=223) | Once or twice a week | **0**·**09 (p=0**·**14)** | **0**·**08 (p=0**·**115)** |
|  | 2 I helped my child to develop friendships by talking to him/her, e.g. I offer advise on how to play with other children, or encourage my child to play with others (n=223) | Once or twice a week | **0**·**08 (p=0**·**18)** | **0**·**1 (p=0**·**05)** |
|  | 3 I asked other parents or children if my child can join in (n=223) | Did not do this | 0·001 (p=0·87) | 0·04 (p=0·42) |
|  | 4 I participated in physical play and leisure activities with my child (n=223) | Nearly daily (5 times or more in the last week) | -0·08 (p=0·23) | -0·05 (p=0·23) |
|  | 5 I supervised my child’s participation in physical play and leisure activities (n=222) | A few times (3-4 times in the last week) | 0·005 (p=0·97) | 0·018 (p=0·7) |
|  | 6 I avoided physical play and leisure activities that I thought were not good for my child. E.g. I avoided taking my child to the toddler play, limited bath play, or cut back on swimming or other activities. (n=223) | Did not do this | 0·07 (p=0·30) | **0**·**10 (p=0**·**06)** |
|  | 7 I stopped a physical play or leisure activity my child has been doing, or a new activity we tried out and decided not to take up (n=223) | Did not do this | 0·005 (p=0·93) | -0·05 (p=0·27) |
|  | 8 I took steps to protect my child in a physical play or leisure situation I considered risky or potentially harmful at the time (n=222) | Once or twice a week | **0**·**11 (p=0**·**095)** | **0**·**07 (p=0**·**15)** |
|  | 9 I chose for us to stay at home rather than go out (n=222) | Did not do this | **-0**·**144 (p=0**·**026)** | **-0**·**08 (p=0**·**1)** |
|  | 11 I decided to register my child in a physical play or leisure activity (n=221) | Did not do this | 0·04 (p=0·52) | 0·06 (p=0·25) |
|  | 12 I made a point to let my child have fun in physical play or leisure activities (n=220) | A few times (3-4 times in the last week) | 0·08 (p=0·22) | 0·09 (p=0·06) |
|  | 13 I followed my child’s lead and let my child to decide what we were going to do (n=220) | A few times (3-4 times in the last week) | 0·04 (p=0·52) | 0·04 (p=0·41) |
|  | 14 I gave my child space to come up with ideas, to expand play, and make it interesting (n=222) | A few times (3-4 times in the last week) | 0·03 (p=0·65) | 0·01 (p=0·78) |
|  | 15 I went on walks, bike rides, or to the park together with my child (n=222) | A few times (3-4 times in the last week) | 0·06 (p=0·38) | **0**·**18 (p<0**·**001)** |
|  | 16 I indicated to other people that adaptations or support may be needed for my child to participate in physical play or leisure (n=222) | Did not do this | 0·03 (p=0·70) | 0·05 (p=0·29) |
|  | 17 I offered to serve as a helper in physical play or activity for my child, or physically helped my child to do activities (n=222) | Once or twice a week | 0·06 (p=0·34) | -0·004 (p=0·93) |
|  | 18 I took my child to physical play sessions or activity clubs, or provided my child access to these types of activities (n=221) | Once or twice a week | 0·05 (p=0·46) | -0·03 (p=0·55) |
|  | 19 I let my child to be more independent in physical play or activity than what they have been before (n=221) | Once or twice a week | -0·002 (p=0·97) | -0·04 (p=0·35) |

**Table S4: Variables included in multivariable regression analysis on the basis that they correlated with physical activity in the univariate analysis**

|  | **Spearman’s rho (p-value)** | |
| --- | --- | --- |
| **Independent Variable** | **Any activity** | **Intense Activity** |
| Age (n=281) | **0**·**18 (0**·**002)** | **0**·**40 (<0**·**001)** |
| IMDDecile (n=277) | 0·05 (0·40) | **0**·**14 (0**·**02)** |
| Recruitment Pathway (n=281) | **-0**·**18 (0**·**002)** | **-0**·**30 (<0**·**001)** |
| Sex (n=280) | **-0**·**07 (0**·**19)** | 0·04 (0·49) |
| Social-cognitive (PEDI-CAT) (n=252) | **0**·**29 (<0**·**001)** | **0**·**57 (<0**·**001)** |
| Mobility (PEDI-CAT) (n=252) | **0**·**35 (<0**·**001)** | **0**·**72 (<0**·**001)** |
| Travel Time to Outdoor Area (n=217) | -0·02 (0·80) | **-0**·**16 (0**·**02)** |
| Main Carer Work Hours (n=221) | **0**·**11 (0**·**10)** | **0**·**11 (0**·**09)** |
| Maximum Daily Temperature (n=281) | -0·07 (0·25) | **-0**·**08 (0**·**17)** |
| Parent Education (n=217) | -0·03 (0·64) | 0·06 (0·36) |

IMD = Index of Multiple Deprivation; PEDI-CAT = Pediatric Evaluation of Disability Inventory Computer Adaptive Testing

**Table S5: Univariable and multivariable linear regression results for HRQoL.**

|  | **Univariable** | | | | **Multivariable (n=92)** | | | |
| --- | --- | --- | --- | --- | --- | --- | --- | --- |
| **Independent Variable** | **Simple Linear Regression** | | | | **Multiple Linear Regression (R^2^=0· 0·65, Adj=0·63)** | | | |
|  | p-value | β | 95% CI | R^2^ | p-value | β | 95% CI | r_s_^2^ |
| Recruitment Pathway (n=100) | **<0**·**001** | **0**·**57** | **[0**·**41, 0**·**73]** | **0**·**32** | **0**·**045** | **0**·**16** | **[0**·**01, 0**·**32]** | 0·5 |
| Age (n=100) | 0·64 | -0·05 | [-0·25, 0·15] | 0·00 | 0·58 | 0·07 | [-0·06, 0·20] | 0·00 |
| Mobility (PEDI-CAT) (n=93) | **<0**·**001** | **-0**·**79** | **[-0**·**92, -0**·**66]** | **0**·**62** | **<0**·**001** | **-0**·**59** | **[-0**·**82, -0**·**35]** | 0·95 |
| Social-cognitive (PEDI-CAT) (n=93) | **<0**·**001** | **-0**·**66** | **[-0**·**82, -0**·**51]** | **0**·**43** | 0·33 | -0·11 | [-0·33,0·10] | 0·67 |
| IMD (Decile) (n=98) | 0·10 | -0·17 | [-0·37, 0·03] | 0·03 | 0·72 | 0·01 | [-0·14, 0·12] | 0·05 |
| Time Active (n=100) | **<0**·**001** | **-0**·**39** | **[-0**·**58,-0**·**21]** | **0**·**15** | 0·52 | -0·05 | [-0·20,0·09] | 0·26 |

IMD = Index of Multiple Deprivation; PEDI-CAT = Pediatric Evaluation of Disability Inventory Computer Adaptive Testing


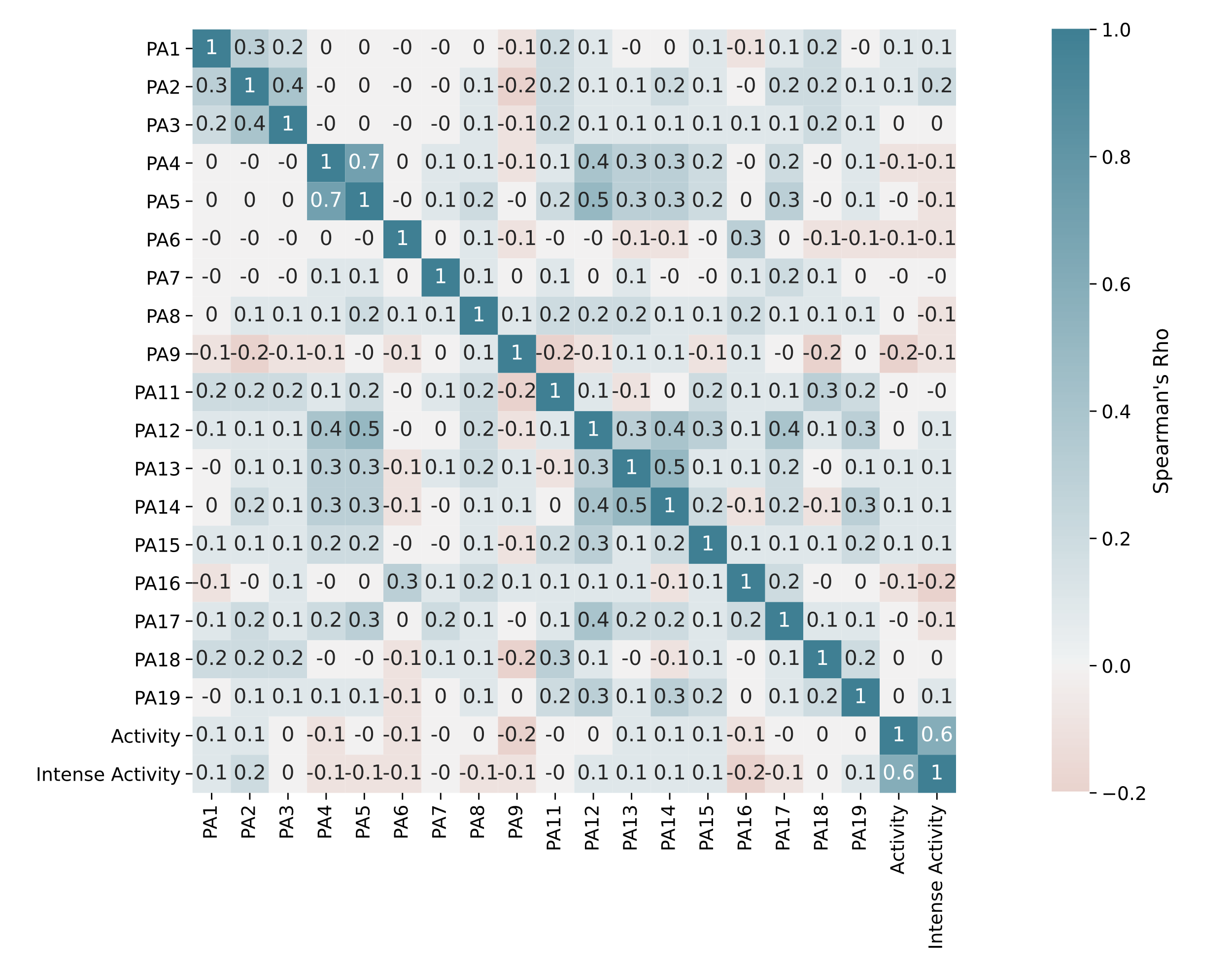


**Figure S1: Pairwise correlation between all parent actions and physical activity participation.**


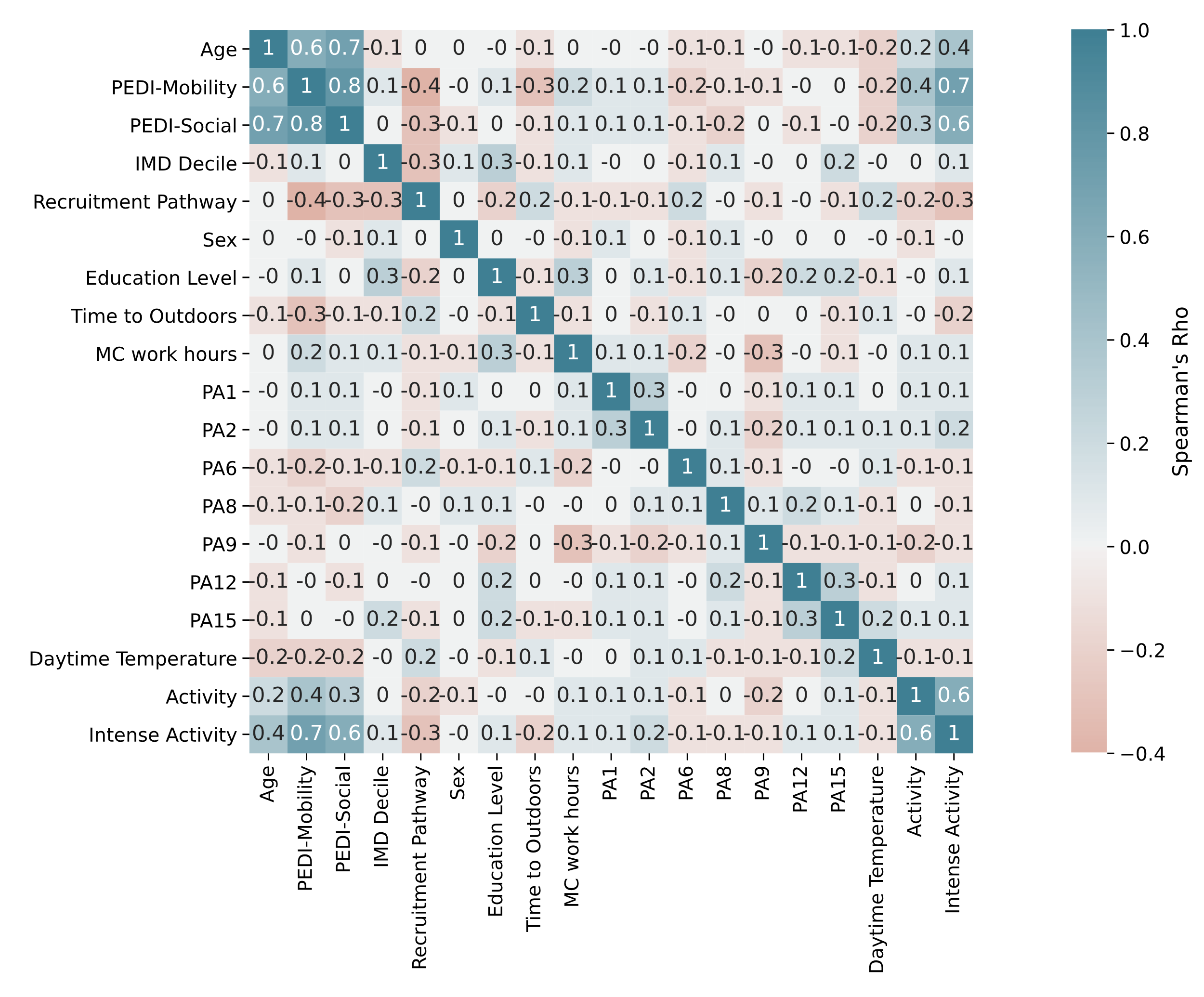


**Figure S2: Pairwise correlation of all variables used in time active multiple regression model**

IMD = Index of Multiple Deprivation; PEDI-CAT = Pediatric Evaluation of Disability Inventory Computer Adaptive Testing; MC = main carer


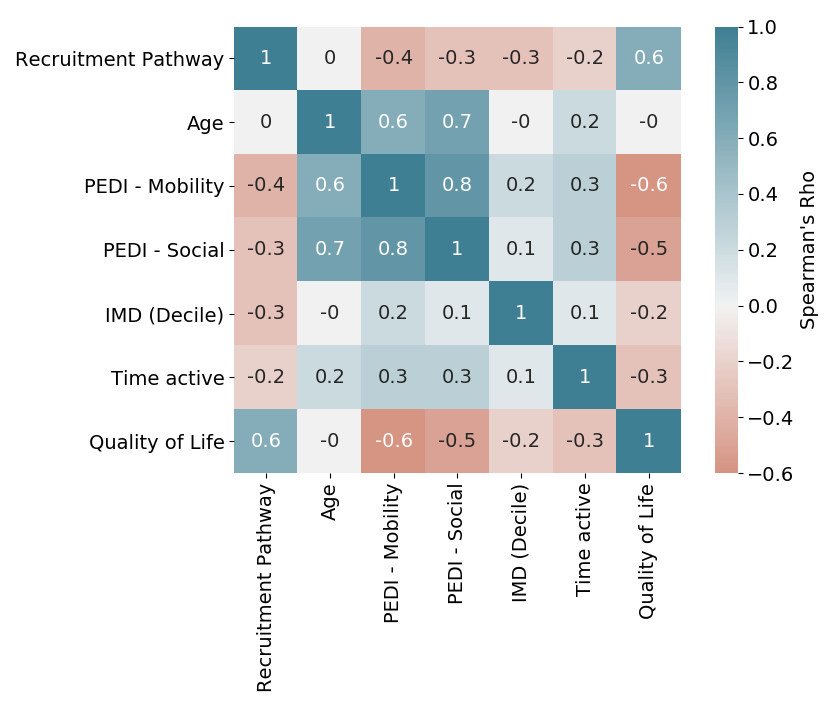


**Figure S3: Pairwise correlation of all variables used in QoL active multiple regression model**

IMD = Index of Multiple Deprivation; PEDI-CAT = Pediatric Evaluation of Disability Inventory Computer Adaptive Testing;
